# Evaluation of Injectate Distribution of the Middle Mental Nerve Block Within the Mandibular Canal in a Cadaveric Canine Model

**DOI:** 10.1177/08987564241293188

**Published:** 2024-10-30

**Authors:** Angus Fechney, David E. Clarke

**Affiliations:** 1 School of Veterinary Science, 6420 Massey University, Palmerston North, New Zealand; 2 Dental Care for Pets, Melbourne, Australia

**Keywords:** middle mental nerve, dogs, regional anesthesia, veterinary dentistry, analgesia

## Abstract

Awareness among veterinarians has increased regarding the need for comprehensive pain relief, but many companion animal veterinarians do not administer regional analgesia pre-emptively during dental procedures. The middle mental nerve (MMN) block desensitizes the ipsilateral mandibular incisor and canine teeth as well as soft tissues rostral to the delivery site. There is little published information on the efficacy of the MMN block in dogs. The objective of this study was to determine injectate distribution within the mandibular canal using a radiopaque contrast media/methylene blue solution. Half a milliliter of solution was injected within the opening of the middle mental foramen using a standard hypodermic syringe and a 25G x 25 mm needle. The course of the injectate was traced both via computed tomography (CT) and, in some cadavers, gross dissection. Post-treatment CT revealed that in 90% of the cases, the contrast diffused at least as far caudally as the mesial root of the third premolar tooth. The injectate was not identified within the canal of 5% of cadaveric mandibles examined. Although the solution used diffused caudally within the mandibular canal when injected using recommended clinical techniques, this may not completely represent the extent of clinical effects experienced in live patients. This technique also confirmed that the needle does not need to be advanced into the mandibular canal to achieve adequate diffusion to at least the mesial root of the third premolar tooth.

## Introduction

In the past 40 to 50 years, dentistry has become an integral facet of veterinary practice.^1^ However, the reasons for neglect of pain associated with any oral condition or dental surgery may vary.

One reason may be associated with a lack of direct teaching and therefore the general principles of dentistry are poorly understood. In one survey, final year undergraduate students almost universally agreed that understanding dentistry was an important part of small animal practice but less than 40% felt they were well prepared once they started in practice. Regional anesthesia is one of the requirements of the Royal College of Veterinary Surgeons (RCVS) but few undergraduates had performed this during their external rotations. The students had least confidence in being able to satisfactorily perform a locoregional block. This may be an indicator of the reduced frequency that this procedure is performed in general practice.^2^

Periodontal disease, tooth fractures, and other painful dental conditions in small animals, such as feline tooth resorption, are undertreated and often not identified as a source of pain.^3^ Typically, therefore, diagnosis in not made until late in the course of the disease.^4^

There may also be concerns associated with the cost of analgesic drugs and of any side effects associated with locoregional blocks in particular.^5^ Additionally, there is concern that administering locoregional blocks around the skull may lead to iatrogenic trauma, e.g., lingual self-trauma, due to the removal of sensory perception of the tongue in the postoperative recovery period after an inferior alveolar block, or due to direct globe trauma when maxillary blocks are performed.^6,7^ As Doctor of Veterinary Medicine (DVM) students in veterinary schools often only receive a minimal education in dental procedures, including the administration of regional nerve blocks and extraction of teeth, it is likely this also contributes to locoregional blocks not being used consistently.^2,8,9^

Very few controlled studies have been published on the prevalence of pre-emptive analgesia for dental procedures in dogs. In human dentistry, the use of local analgesia, in conjunction with general anesthesia, is a common treatment regime.^10^ The use of a balanced anesthetic protocol is a widely used concept in companion animal anesthesia.^11^

Regional anesthesia should be considered a vital adjunct to any oral or dental procedure that will cause pain. The mechanism of action for local anesthetics is by blocking the inward movement of Na^+^ into neurons through voltage-gated Na^+^ channels. This halts propagation of the electrical signal by impeding membrane depolarization and nerve excitation and conduction.^12^

Inhalant anesthetics are routinely used to maintain anesthesia and allow the practitioner to perform dental procedures on veterinary patients. Even when used at minimum concentrations, these drugs are associated with dose-dependent adverse cardiorespiratory effects.^13^ A decrease in blood pressure is not uncommon and is associated with a decrease in cardiac output.^11^ The safety of general anesthesia is therefore improved if a combination of techniques are available to decrease the inhalation anesthetic needed for general anesthesia.^14^

It has been shown that when used in combination with general anesthesia, regional nerve blocks with local anesthetics decrease minimum alveolar concentrations of gas anesthetics, and postsurgical pain perception.^15^ Desensitization of the middle mental nerve (MMN) is a regional block technique previously described in the literature.^13,16‐18^

The MMN is the terminal branch of the mandibular nerve after exiting the middle mental foramen. The mandibular nerve is 1 of 3 branches of the trigeminal nerve (cranial nerve V) providing sensory innervation to the teeth and associated soft tissues of the face. The 3 branches are the ophthalmic, maxillary, and mandibular nerves.^13^ The mandibular branch courses along the lingual surface of the ramus and enters the mandibular canal where it becomes the inferior alveolar nerve (IAN) as it courses rostrally in the mandibular canal. The IAN provides sensory innervation to the teeth in the ipsilateral mandible and the mental nerves provide sensory innervation to the lower lip and rostral intermandibular region.^19‐21^ Anesthetic injected into the middle mental foramen reaches the mandibular canal and rostral alveolar nerve branch of the IAN. Affected tissues include the ipsilateral mandibular incisors, canine, first premolar teeth, and adjacent soft tissues.^17^ In the dog, the middle mental foramen is ventral to the alveolar crest, dorsal to the ventral cortex of the mandible and immediately caudal to the labial frenulum.^20^

Desensitization of the incisive regions and rostral regions of the mandible was not reliably demonstrated in a recent study. The area of desensitized tissues was smaller than expected and highly variable within the study group. The block was most consistent in desensitizing the second to fourth premolar teeth. The soft tissues were not dependably blocked, with one region receiving analgesia in more than half of the 7 dogs.^22^

When considering the neuroanatomy of this area, it seems plausible that if a local anesthetic agent can be placed in the rostral portion of the mandibular canal via the middle mental foramen, the IAN could be desensitized from the point to which the injectate diffuses caudally along the length of the nerve rostral to this point.

The objectives of this study were to determine injectate distribution within the mandibular canal using a combined dye and radiopaque contrast media solution in cadaveric dogs and to determine the extent of methylene blue (MB) dye distribution when no radiopaque contrast was identified within the mandibular canal.

## Materials and Methods

Ethically sourced cadavers were used in this study. The cadavers were not euthanized for the purposes of research or teaching, therefore, ethical approval was not required under the New Zealand Animal Welfare Act (1999).

Twenty dog cadavers were available and had been previously humanely euthanized for reasons other than this study. The cadavers had been stored at 20^o^C. Once thawed, the heads were examined for signs of maxillofacial trauma or other deformity that would exclude them from the study. All heads were confirmed to have complete adult dentition. Sex and weight were not available. One dolichocephalic dog was present, and all other heads were considered mesaticephalic. Heads were removed from the bodies at the first cervical vertebrae and the heads were individually stored in plastic bags at 4^o^C.

Computed tomographic (CT) imaging was used for all specimens on a 16-slice CT scanner^a^. Images were acquired in 0.5 mm transverse slices pre- and postcontrast injection and reconstructed into 2 × 1 mm overlap bone algorithms. The images were acquired in sternal recumbency (lower jaw placed on the imaging table). Individual preloaded syringes^b^ containing 0.25 mL of iohexol^c^ mixed with 0.25 mL of 1% methylene blue^d^ (MB) were placed into the radiographic contrast warmer^e^ at 39°C until required.

**Figure 1.**
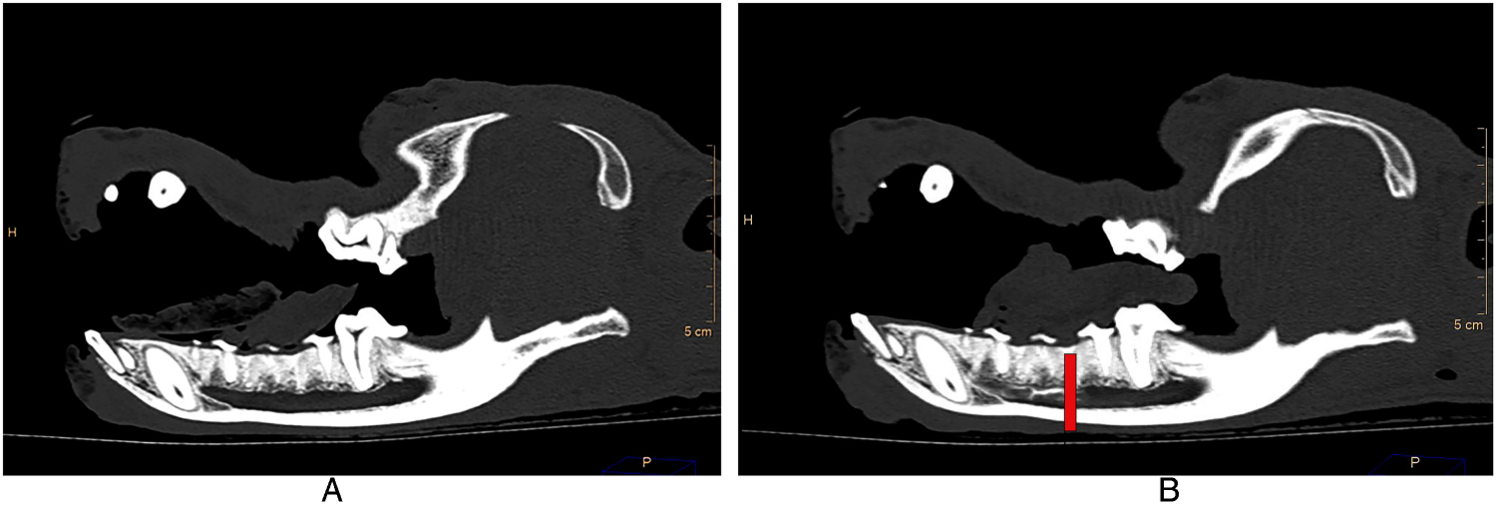
Sagittal view of CT image. (A) Precontrast and (B) postcontrast showing distribution of the contrast to the level of the distal root of the third premolar tooth (red line).

A precontrast CT of the head was acquired (Figure 1A). The MNN block was then performed as has been described previously.^18^ The depression of the middle mental foramen was palpated ventral to the second premolar buccal to the point of the apex of the mandibular canine and just caudal to the labial frenulum. The syringe with a 25G x 25 mm needle^f^ was inserted through the mucosa of the mesial border of the lip frenulum and directed ventrocaudally so that the tip was positioned 2 to 4 mm inside the middle mental canal. As the needle was placed into the canal, a finger was placed firmly over the mucosal margin of the middle mental foramen to increase the probability of the injectate dispersing caudally into the mandibular canal. Half a milliliter of 1:1 iohexol:MB injectate was then slowly injected over 10 s, while palpating for any ballooning of the soft tissue around the foramen that might indicate the needle might have become displaced from within the canal. If any fluid was palpated caudal to the middle mental foramen, the needle tip was withdrawn 3 to 4 mm and then redirected toward the foramen and the remaining volume of injectate given.

The right-side mental block was performed initially and then followed directly by blocking the left mandible. Ten minutes after finishing the right block the postcontrast CT was acquired (Figure 1B). All injections were performed by the same author (AF). All mandibles were assessed radiographically to determine the caudal extent of the iohexol within the mandibular canal. This was visually determined to be the point at which the contrast was just beginning to dissipate. The tooth root that was just mesial to this point was recorded for both the left and right mandibles.

**Figure 2.**
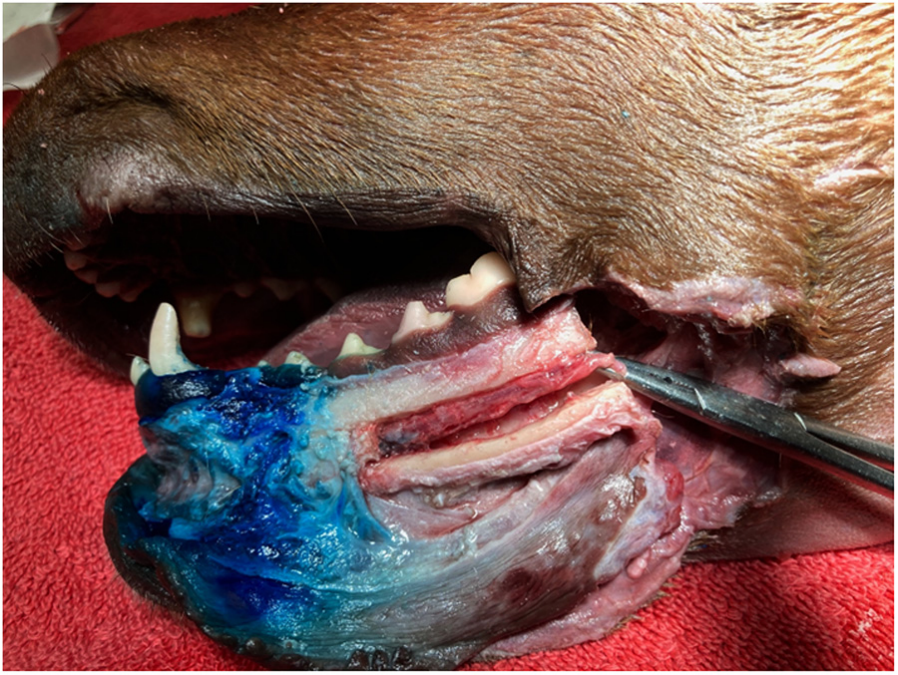
Case #11 showing extent of methylene blue in soft tissue around the middle mental foramen and confirming slight staining ventrally on the neurovascular bundle within the mandibular canal.

Gross dissection of 4 cadavers was performed to determine the placement of the MB in the heads that showed indeterminate caudal diffusion of the contrast media. This included 2 mandibles where no iohexol was evident within the mandibular canal and 2 mandibles where diffusion was only seen as far as the distal root of the second premolar tooth (Figure 2).

The dissection was performed by incising longitudinally around the mucogingival line with a scalpel. Using a periosteal elevator, the soft tissue was reflected ventrally along the length of the dental arcade exposing the entire surface of the buccal surface of the mandible. Distribution of the MB was noted and then a tungsten carbide bur in a high-speed hand piece was used to cut a longitudinal channel in the bone at the most dorsal and ventral aspect of the mandibular canal from the level of the distal aspect of the second premolar through to the distal aspect of the first mandibular molar. This produced 2 parallel cuts with the bone “window” still attached both mesially and distally. The volume of water coolant associated with the high-speed handpiece was reduced in order to decrease the potential for spread of the MB further distally within the canal along with careful sectioning of the bone only just as deep as the canal itself. A vertical section through the mandible just distal to the mandibular first molar allowed visualization of the neurovascular bundle (IAN, artery, and vein) and allowed the bone window to be lifted exposing the more rostral portion of the neurovascular bundle.

The soft tissue around the mental foramina, the mandibular canal and the IAN were examined circumferentially for evidence of staining.

## Results

**Figure 3.**
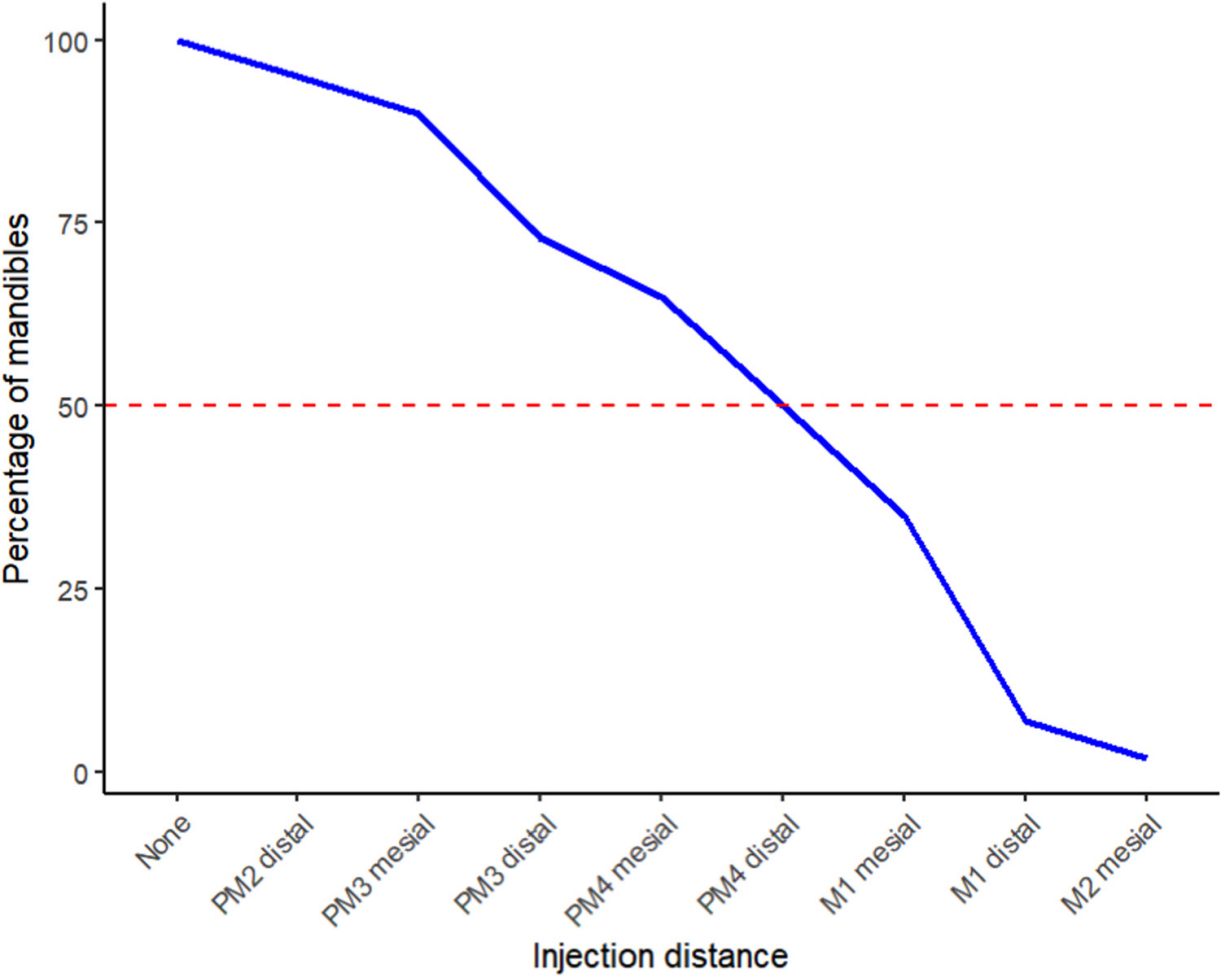
The line graph shows the caudal extent of the injectate measured as a percentage of mandibles relative to the tooth roots. Abbreviations: PM, premolar tooth; M, molar tooth.

The 20 heads provided 40 mandibles for examination. Contrast was demonstrated within the mandibular canal in 38 mandibles. Two of the 40 mandibles examined showed no evidence of either iohexol or MB within the mandibular canal. The point at which the contrast dissipated on visual examination of the CT images was designated as the most caudal distribution point within the mandibular canal. This location was then correlated to the nearest mandibular tooth root rostral to this point. Figure 3 represents the caudal extent of the injectate measured as a percentage of mandibles relative to the tooth roots where the molars are to the furthest right of the graph.

The contrast diffused to the canine tooth in 38 of 40 (95%), first premolar in 38 of 40 (95%), mesial root of second premolar in 38 of 40 (95%), distal root of second premolar in 36 of 40 (90%), mesial root of third premolar in 36 of 40 (90%), distal root of third premolar in 30 of 40 (75%), mesial root of fourth premolar in 27 of 40 (67.5%), distal root of fourth premolar in 20 of 40 (50%), mesial root of first molar in 15 of 40 (37.5%), distal root of first molar in 5 of 40 (12.5%), mesial root of second molar in 2 of 40 (5%). In no heads did the contrast reach the distal root of second molar 0 of 40 (0%). Two of the 40 mandibles examined showed no evidence of either iohexol or MB within the mandibular canal. In these 2 cases there was MB staining caudal to the middle mental foramen in the submucosa. Two further CT studies showed caudal distribution of the injectate only as far as the mesial root of the second premolar in 2 of the mandibles. This was corroborated by MB being evident in and around the middle mental foramen but not diffusing any further caudally in one case and only being present ventrally on the IAN at the level of the third premolar in the other case.

**Figure 4.**
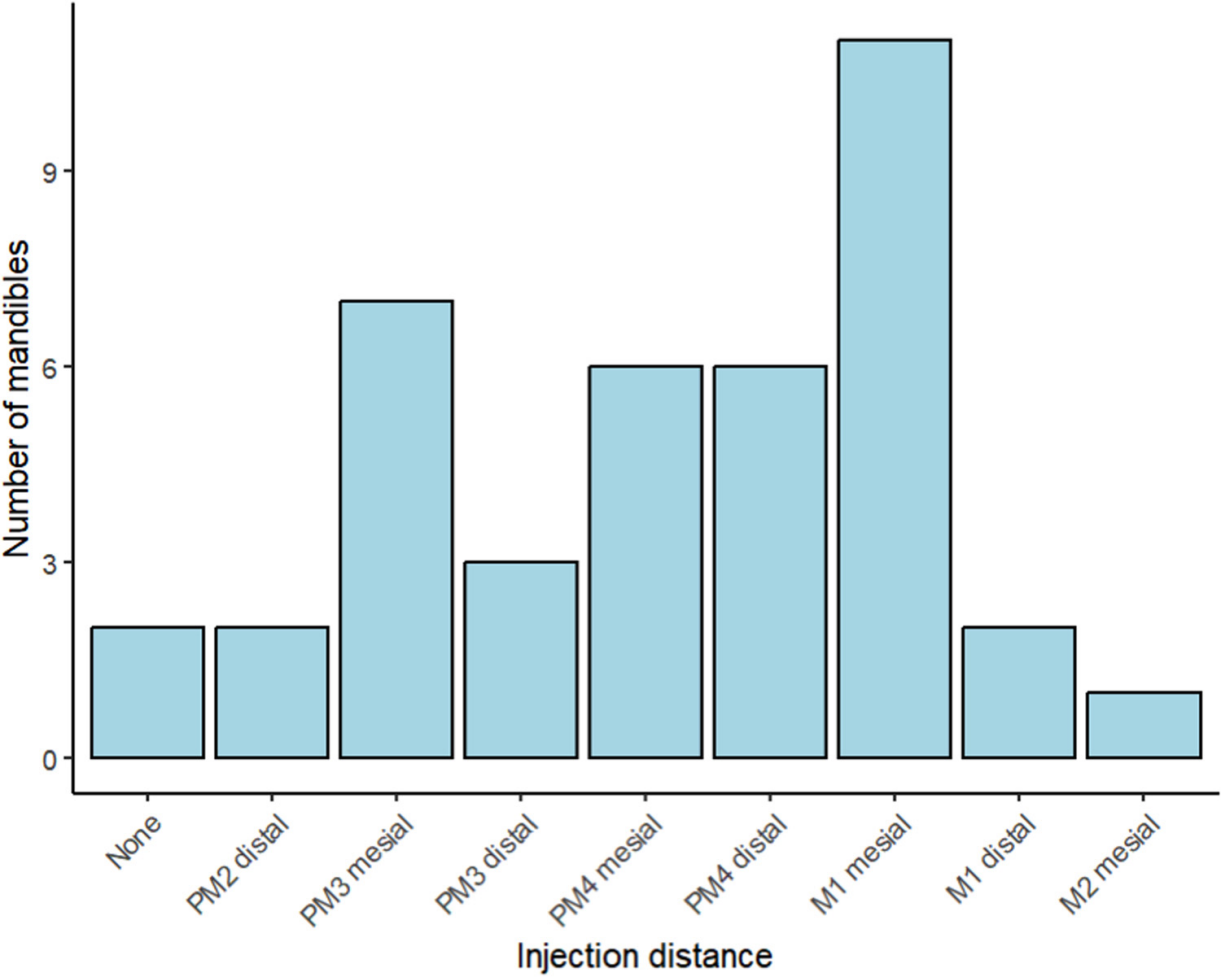
The most caudal extent of the injectate dispersion, in each individual mandible, through the mandibular canal relative to the tooth roots as identified on the postcontrast computed tomography images. The height of the bars indicates the number of mandibles that were in each tooth root category. Abbreviations: PM, premolar tooth; M, molar tooth.

Figure 4 depicts the most caudal extent of the injectate dispersion, in each individual mandible, through the mandibular canal relative to the tooth roots as identified on the postcontrast CT images. The height of the bars indicates the number of mandibles that were in each tooth root category.

## Discussion

The results of this study confirm that solution injected into the middle mental foramen can distribute caudally inside the mandibular canal.

A previous clinical study of 7 dogs had shown that the MMN block had not consistently blocked the soft tissue or dental structures of the rostral mandible.^22^ One of the aims of the current study was to provide confirmation that an injected volume of fluid would diffuse sufficiently caudally to theoretically provide anesthesia to more rostral structures. From a neuroanatomy perspective, to effectively desensitize the canines and incisors, the local anesthetic agent must be deposited into the middle mental foramen and infraorbital canal in the mandible and maxilla, respectively. When the anesthetic is placed outside the canal, it is likely that only soft tissue structures will be anesthetized.^15,16^

One of the factors determining nerve impulse transmission blockade is the length of nerve exposed to a local anesthetic agent. Exposure lengths of 3 nodes of Ranvier (approximately 6 mm in length) ensures approximately 50% block of impulses in single nerve fibers.^23^

The volume of solution chosen for this study was 0.5 mL as this is an approximate volume indicated in one review for any dog larger than a small dog size^5^ and using 0.5% solution would be well under the maximum dose of bupivacaine for a 10 kg dog (maximum dose of 0.5% bupivacaine = 2 mg/kg×10 kg = 20 mg which equates to a volume of 4 mLs total for all blocks during a procedure). A smaller or larger volume may have produced different results, providing a smaller or larger area of distribution, respectively. Another author suggests the formula 0.13 mL/kg^2/3^ to calculate the volume for injection but it is acknowledged that this does not consider the variation in size of the mandibular canal between breeds.^15^ Using this formula equates to 0.6 mLs in a 10 kg dog. This variation between breeds is also a limitation of this study as it would be ideal if the canals tested were more uniform. An attempt was made to reduce this variability by selecting dogs with a certain head type.

The results of the current study show that the injectate diffused caudally to at least the mesial root of the third premolar in 90% of mandibles and as caudally as the mesial root of the fourth premolar in at least two-thirds of mandibles. This may indicate that in a live patient, the inferior alveolar branches supplying the teeth may in fact be desensitized to the same level. This does not correlate well with the results from the previous study^22^ but some of the variation may simply be differences between a cadaveric study and a live study. This is therefore one of the limitations of this current study as there is an assumption that physical diffusion of the injectate equates to anesthesia at the same distance caudally in the mandibular canal. This can only be tested in live studies. In the current study, imaging was performed after 10 min as opposed to 30 to 45 min elapsing before the noxious stimuli was administered to the live patients in the previous study. Despite the extended timeframe, given the duration of effect of bupivacaine: 180 to 480 min,^5^ it seems likely that the areas of the rostral oral cavity would have remained desensitized in the previous study.

The viscosity of the injectate solution may have influenced the extent of distribution but despite the warming, it is likely the viscosity of the mixed injectate (iohexol and MB) was greater than a pure bupivacaine mixture, as used in the previous study.^22^ The viscosity of iohexol is 3.3 mPa S at 37^o^C^24^ as opposed to bupivicaine which is likely to be no greater than 1 mPa S at 37^o^C (water is 0.6913 mPa S at 37^o^C^25^). The previous study used an injection rate of 0.2 mL/s whereas in the current study 0.5 mL was administered over approximately 10 s, partly as a result of the increased viscosity of the solution. In this study, a 25G x 25 mm needle was used, as the gauge is consistent with what has previously been recommended for medium-large dogs.^15^ Other than use of a dental syringe and needle, a 25G needle is also the narrowest gauge hypodermic needle available locally that includes lengths greater than 13 mm for larger dogs.

In the previous study, 7 mandibles were injected with bupivacaine and then noxious stimuli were applied after 30 to 45 min.^22^ Four other mandibles had a mixture of bupivacaine and iohexol injected to check correct placement of the injectate. All 4 of these latter mandibles showed diffusion of contrast radiographically caudally in the mandibular canal but no noxious stimuli were applied to these mandibles. It is possible that inadvertent injection in the caudal mental foramen may help explain more consistent desensitization further caudally in the previous study particularly as the volume of injectate used was smaller.

Post contrast CT was performed in the current study at 10 min after the first injection. This time period is considered the upper limit for onset of action for bupivacaine and ropivacaine, 2 commonly used local anesthetic agents.^5^ When investigating ultrasound guided deposition of bupivacaine and lidocaine around the brachial plexus in cats, one author reported a measured response after 15 min.^26^

The timing of analgesic administration is important, as anesthesia does not necessarily equate with analgesia. It has been shown that analgesics administered before inducing pain, termed “pre-emptive analgesia” greatly reduces the degree of pain the central nervous system processes and the resultant postoperative perceived pain.^26‐28^ The entire concept of “pre-emptive analgesia” rests on the belief that one is blocking nociceptive stimuli from forward transmission to the central nervous system. If this forward transmission is not blocked, then central sensitization (sometimes referred to as “wind-up”) may occur. The response occurs as a result of prolonged or noxious stimuli at the level of the dorsal horn which in turn results in amplification of subsequent signals to the brain that are not related to the intensity or duration of the initial stimulus. Though the response is multifactorial, N-methyl D-asparate receptors are critical. As a result, any subsequent painful event is likely to be perceived as more painful.^29^ This central hypersensitivity results in a heightened response to subsequent afferent inputs, which lasts between 10 and 200 times the duration of the initiating stimulus.^30^ This induces neural and behavioral changes that persist even when inputs from the injured region are subsequently blocked by local anesthetic.^31^ One of the ongoing aims of this and subsequent similar studies on live patients is to provide further options for clinicians for employing pre-emptive analgesia.

This study used an equal mix of MB with iohexol for 2 reasons. Firstly, it confirmed the accuracy of the iohexol when it wasn’t readily apparent on CT. Secondly, to act as a diluent, because iohexol is significantly more viscous than local anesthetic agents as discussed previously. Previous studies substituting MB and iohexol for a local anesthetic agent have used 1:1 MB:iohexol.^32,33^ This, however, is a limitation of the study as even the warming of the syringes and dilution of the iohexol would not give the injectate the same viscosity as a local anesthetic agent and this would therefore affect the distribution pattern within the mandibular canal. Other limitations associated with distribution are the uncontrolled temperature variation of the heads and associated soft tissue in and around the mandibular canal. Though the thawing process was as uniform as possible, there was no standardization of the temperature of the heads and no method to determine the quality of tissues within the mandibular canal just prior to injection.

Two of the 40 mandibles examined showed no evidence of either iohexol or MB within the mandibular canal. In these 2 cases there was MB staining caudal to the middle mental foramen in the submucosa and therefore it was operator error that had caused the failure of the injectate to diffuse caudally in the mandibular canal. All injections were administered by the same author (AF) to ensure consistency. This author normally uses a reusable self-aspirating dental syringe with a more flexible, narrower gauge needle but wanted to use a disposable syringe and hypodermic needle as these are cited in other references.^5,22^

When needed, redirection of needle was warranted along with light pressure over the needle as the solution is injected and again for 20 s post needle withdrawal as these procedures mimic the clinical situation and are described in other texts.^15^ The exact position of the needle tip, whether it be in soft tissue, fat or a fascial plane, may have a bearing on solution distribution in individual heads. All these factors may have a bearing on effectiveness in a live patient. Due to the nature of the injection, and the precise location of the needle within the canal foramen adjacent to the neurovascular bundle, it is critical that the needle used is short and as narrow gauge as possible to prevent neurovascular trauma.^15,16,18^

Whenever injecting into a foramen containing a neurovascular bundle, there is increased risk of neurovascular trauma. This is exacerbated when the foramen is smaller.^34^ As a needle needs to be advanced into the middle mental foramen to effect a MMN block, some authors state that the increased risk of soft tissue trauma outweighs any benefits of regional anesthesia in the rostral mandible so recommend the IAN block if extracting any mandibular teeth.^5,35^ There is a risk of anesthetizing the lingual nerve when performing the IAN block. When the lingual nerve is desensitized, there is a risk of lingual trauma as the animal recovers from general anesthesia.^5^ This risk is likely increased when bilateral IAN desensitization is necessary. With the risk of causing inadvertent self-trauma to the tongue, some practitioners may decide against any local anesthesia in the caudal mandible. However, if an MMN block is effective then this risk can be mitigated for any rostral mandibular surgery.

This study confirmed the distribution of solution caudally into the mandibular canal, with 95% reaching the mesial root of the second premolar tooth and showed an overall even distribution of solution from the mesial root of the second premolar to the mesial root of the second molar. The authors believe that when local anesthesia is used as described for an MMN block in an anesthetized patient, all teeth and most soft tissues mesial to the second premolar would be consistently desensitized. Further studies to confirm the exact areas of desensitization in live patients are necessary to determine how this distribution of contrast and dye relates to the clinical effect in living patients.

Additional work using brachycephalic models would also be beneficial.
